# The Association Between Aspartate Transaminase to Alanine Transaminase Ratio and Perioperative Ischemic Stroke in Patients With Diabetes: A Retrospective Cohort Study

**DOI:** 10.1111/cns.70223

**Published:** 2025-01-21

**Authors:** Siyuan Liu, Binbin Wang, Libin Ma, Huikai Yang, Min Liu, Yuxiang Song, Zhikang Zhou, Jingsheng Lou, Daming Zhou, Jiangbei Cao, Yanhong Liu, Weidong Mi, Yulong Ma

**Affiliations:** ^1^ Department of Anesthesiology The First Medical Center of Chinese PLA General Hospital Beijing China; ^2^ National Clinical Research Center for Geriatric Diseases Chinese PLA General Hospital Beijing China; ^3^ Department of Anesthesiology Affiliated Hospital of Nantong University Nantong China; ^4^ Department of Anesthesiology Beijing Tongren Hospital, Capital Medical University Beijing China; ^5^ Department of Infection Control JILIN Cancer Hospital Jilin China

**Keywords:** alanine transaminase (ALT), aspartate transaminase (AST), De Ritis ratio, perioperative ischemic stroke, type 2 diabetes mellitus

## Abstract

**Background:**

Patients with diabetes are at a high risk for perioperative ischemic stroke (PIS). The use of biomarkers to identify high‐risk patients and predict PIS may provide considerable reference value in clinical decision‐making. The aspartate transaminase/alanine transaminase ratio (De Ritis ratio) has been proven to be associated with specific diabetic complications. However, the association between the De Ritis ratio and PIS has not been evaluated in this population. This retrospective cohort study aimed to evaluate the association between the preoperative De Ritis ratio and PIS in patients with type 2 diabetes undergoing noncardiovascular surgery.

**Methods:**

Data from surgical patients were collected from January 2008 to August 2019. A total of 27,643 patients with type 2 diabetes mellitus (DM) undergoing noncardiovascular surgery under general anesthesia were screened. The optimal De Ritis ratio cutoff value was identified using the receiver operating characteristic (ROC) curve. Logistic regression models were used to evaluate the association between the preoperative De Ritis ratio and PIS. Propensity score matching (PSM), sensitivity analyses, and subgroup analyses were performed to further validate the robustness of this association.

**Results:**

A total of 151 patients experienced PIS. A De Ritis ratio ≥ 1.04 was associated with an elevated risk of PIS after adjusting for baseline characteristics (OR [95% CI]: 2.25 [1.59–3.21]; *p* < 0.001), intraoperative parameters (2.50 [1.80–3.49]; *p* < 0.001), and all confounding variables (2.29 [1.61–3.29]; *p* < 0.001). In the propensity score‐matched cohort, the association between the De Ritis ratio and PIS remained significant (2.04 [1.38–3.05]; *p* < 0.001). These associations were also consistently maintained in the sensitivity and subgroup analyses.

**Conclusions:**

An elevated De Ritis ratio is strongly associated with a higher risk of PIS in patients with type 2 DM undergoing noncardiovascular surgery. This may provide additional information on PIS risk assessment in patients with type 2 DM undergoing noncardiovascular surgery.

## Graphical abstract

Individuals who developed perioperative ischemic stroke (PIS) exhibited a higher aspartate transaminase to alanine transaminase ratio (De Ritis ratio). The odds of PIS were significantly increased for patients with a De Ritis ratio ≥ 1.04 in the multivariable logistic regression, propensity score matching (PSM) analysis, and several sensitivity analyses and subgroup analyses.

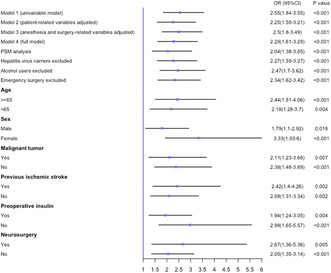

**Abbreviations**
ALT: alanine transaminase
ASA: American Society of Anesthesiologists
AST: aspartate transaminase
BMI: body mass index
CHD: coronary heart disease
CI: confidence interval
COPD: chronic obstructive pulmonary disease
DM: diabetes mellitus
FPG: fasting plasma glucose
HF: heart failure
IQR: interquartile range
MI: myocardial infarction
NAFLD: nonalcoholic fatty liver disease
OR: odds ratio
PIS: perioperative ischemic stroke
PS: propensity score
PSM: propensity score matching
PVD: peripheral vascular disease
ROC: receiver operating characteristic

## 1 Background

Perioperative stroke is commonly defined as a new‐onset cerebrovascular accident occurring within 30 days after surgery, the majority of which appears to be ischemic [1, 2, 3]. The incidence of perioperative ischemic stroke (PIS) ranges from approximately 0.1%–1.9% in noncardiac surgery, depending on risk factors [4, 5]. Despite its low incidence, PIS has serious consequences, including prolonged hospital stay, substantial disability, and high mortality rates.

Risk factors for PIS have been identified in recent years, among which diabetes mellitus (DM) is a crucial contributor [6]. The prevalence of DM is 11.2% in mainland China and may be still increasing [7]. The reported total incidence of PIS is 0.22% in the whole surgical population and 0.53% in diabetic patients [8, 9], indicating an increased risk of PIS in diabetic surgical patients. Furthermore, trends in PIS are less favorable in patients with DM than in those without DM [10]. Early prediction of PIS in patients with DM may be beneficial for ensuring that they receive closer monitoring during the perioperative period. Hence, it is necessary to find easily assessable biochemical indicators to better predict the occurrence of PIS in patients with DM. However, few studies have reported biochemical indicators of PIS in surgical patients with DM.

Aspartate transaminase (AST) and alanine transaminase (ALT) are two routinely available biochemical values for surgical patients that derive the AST/ALT ratio (De Ritis ratio). The De Ritis ratio has been used for viral hepatitis diagnosis and nonalcoholic steatohepatitis discrimination [11, 12]. Previous studies have indicated that the De Ritis ratio is negatively associated with type 2 DM because serum ALT levels increased with the progression of impaired fasting glucose and diabetes [13, 14]. Generally, a De Ritis ratio < 1 suggests nonalcoholic fatty liver disease (NAFLD), and a reversed De Ritis ratio > 1 is a significant indication of hepatic fibrosis or even cirrhosis [12, 15]. The De Ritis ratio has also been used as a prognostic factor for acute myocardial infarction [16]. Moreover, an elevated De Ritis ratio has been associated with high mortality after cardiac arrest and poor outcomes after ischemic stroke [17, 18]. A recent study suggested that the De Ritis ratio is independently correlated with diabetic nephropathy and inflammatory cytokine levels in type 2 diabetic patients [19]. However, to the best of our knowledge, the association between the De Ritis ratio and the risk of PIS in patients with type 2 DM undergoing noncardiovascular surgery has not been evaluated.

In the current study, the association between the De Ritis ratio and PIS risk in patients with type 2 DM undergoing noncardiovascular surgery was investigated. We hypothesized that an elevated De Ritis ratio is associated with an increased risk of PIS in patients with type 2 DM who underwent noncardiovascular surgeries.

## 2 Methods

### 2.1 Study Design

This cohort study was approved by the Medical Ethics Committee of the First Medical Center of the Chinese People's Liberation Army (PLA) General Hospital (reference number: S2022‐607‐01), and the requirement for informed consent was waived. The current research adhered to the Strengthening the Reporting of Observational Studies in Epidemiology (STROBE) guidelines. All patients who underwent surgery were recruited between January 2008 and August 2019. The inclusion criteria were patients aged 18 years or older, noncardiovascular surgery, and general anesthesia with a surgery length above 1 h. The exclusion criteria were ASA classification of V or above, no diabetes diagnosis, type 1 DM, and missing data for any variables. For patients who underwent multiple procedures within the study period, the first surgery was used as an index procedure. Finally, 27,643 participants were included in this study.

### 2.2 Data Collection

All clinical data were retrieved electronically from a central database. Baseline characteristics and intraoperative parameters were collected. Age, sex, body mass index (BMI), American Society of Anesthesiologists (ASA) classification, tobacco use, alcohol use, hypertension, coronary heart disease (CHD), heart failure (HF), previous myocardial infarction (MI), arrhythmia, chronic obstructive pulmonary disease (COPD), renal insufficiency (defined by creatinine > 177 μm/L), peripheral vascular disease (PVD), malignant tumor, previous ischemic stroke, hepatitis virus carriage condition, preoperative fasting plasma glucose (FPG), preoperative hemoglobin, preoperative platelet, preoperative albumin, preoperative total bilirubin, insulin medication, and preoperative anticoagulants were defined as baseline characteristics. The urgency of surgery, surgical category, surgery length, crystalloid infusion, colloid infusion, and intraoperative blood product usage were defined as intraoperative parameters.

Routine laboratory values were determined based on the results from the central laboratory during preoperative evaluation, and the results closest to the date of operation were used. The optimal cutoff for the De Ritis ratio was identified using a receiver operating characteristic (ROC) curve (Figure S1). PIS was defined as new‐onset cerebral infarction during the hospital stay. The diagnoses of PIS were confirmed by the neuroimaging combined with clinical evidence of cerebrovascular ischemia, identified through ICD9/ICD10 diagnosis codes (Table S1).

### 2.3 Confounding Variables

All the aforementioned variables were included in Model 1, which is a univariate model for crude analysis. Baseline characteristics, including age, sex, ASA classification, tobacco use, alcohol use, hypertension, CHD, HF, previous MI, PVD, previous ischemic stroke, preoperative FPG, preoperative hemoglobin, preoperative platelet, preoperative albumin, preoperative insulin, and preoperative anticoagulants, were adjusted in Model 2. Intraoperative parameters, including urgency of surgery, surgical category, surgery length, and intraoperative blood products usage, were adjusted in Model 3. All the variables were adjusted in Model 4 as a full model.

### 2.4 Propensity Score Matching (PSM)

We performed PSM to adjust for between‐group differences. Variables with a standardized mean difference (SMD) over 0.10 between groups were included in the PSM model. In the PSM analysis, the propensity score (PS) was estimated using a multivariable logistic regression model, in which the De Ritis ratio was the dependent variable and confounding variables were explanatory variables. Confounding variables, including age, sex, BMI, ASA classification, tobacco use, alcohol use, preoperative FPG, preoperative hemoglobin, preoperative albumin, preoperative total bilirubin, urgency of surgery, intraoperative crystalloids, and blood product usage, were matched in PSM. Participants with De Ritis ratio ≥ 1.04 and De Ritis ratio < 1.04 were matched by PS at a 1:1 ratio with a caliper of 0.05.

### 2.5 Statistical Analysis

The Shapiro–Wilk test was used to test the normality of the distribution of continuous variables. Continuous variables are summarized as median and interquartile range (IQR) and are compared using the *t*‐test or Mann–Whitney *U* test. Categorical variables are summarized as the number and percentage of patients and are compared using the chi‐squared test or Fisher's exact test. A univariate analysis was performed, and candidate variables with *p* < 0.05 were included in the multivariable model.

Multivariable logistic regression was used to determine whether the De Ritis ratio was independently associated with PIS risk in patients with type 2 DM. The PIS was modeled as the dependent variable, and the De Ritis ratio was modeled as an independent variable.

We conducted a sensitivity analysis by excluding subjects who were positive for HBV and/or HCV, alcohol users, and those who underwent emergency surgery. We also conducted a subgroup analysis to further investigate whether the association between the De Ritis ratio and PIS differs among selected subgroups. Statistical analysis was performed using SPSS (version 26.0, IBM Corp, USA) and R (version 4.0). All statistical tests being two‐sided, *p* < 0.05 was considered statistically significant. Odds ratios (OR) with 95% confidence intervals (CI) are reported for all models.

## 3 Results

### 3.1 Cohort Characteristics

**FIGURE 1:**
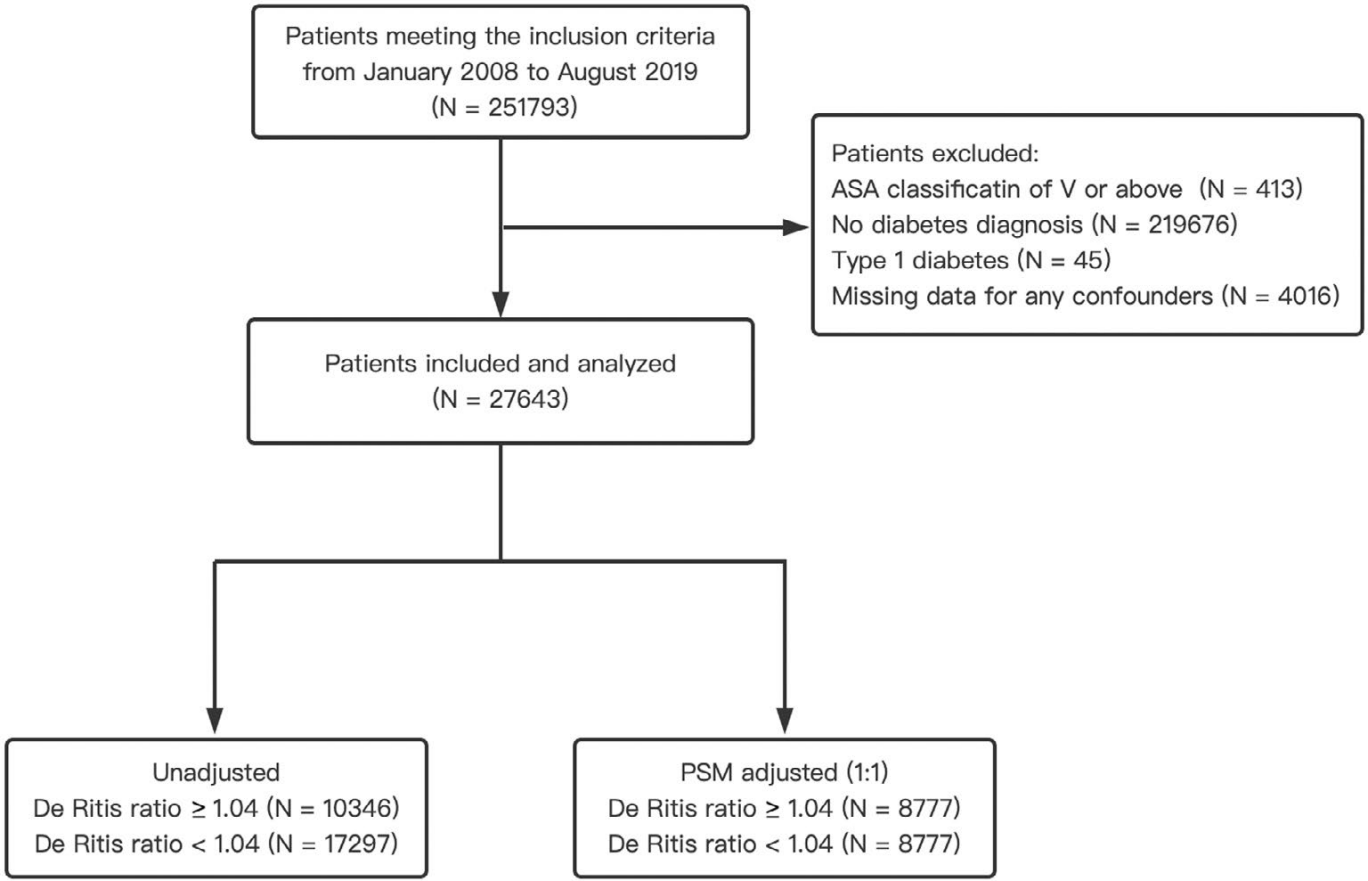
Patient flow diagram. ASA, American Society of Anesthesiologists; PSM, propensity score matching.

The study cohort consisted of 27,643 patients with type 2 DM after the application of inclusion criteria and exclusion criteria. A patient flow diagram for this study is shown in Figure 1. The overall incidence of PIS was 0.55% (*N* = 151). Among 27,643 patients with type 2 DM, 32.5% (8990) of them were aged ≥ 65 years 57.8% (15,994) were male, 51.8% (14,308) had malignant tumors, 5.5% (1529) had a history of ischemic stroke, 51.4% (14,214) were on insulin medication, and 8.6% (2382) underwent neurosurgery.

### 3.2 Association Between De Ritis Ratio and PIS

Patients were then divided into low (< 1.04, *n* = 17,297, 62.6%) and high (≥ 1.04, *n* = 10,346, 37.40%) De Ritis ratio groups according to the ROC curve‐defined optimal cutoff. PIS occurred in 60 (0.35%) patients in the low De Ritis ratio group and 91 (0.88%) patients in the high De Ritis ratio group. Descriptive statistics comparing patients in the low De Ritis ratio group to those in the high De Ritis ratio group are shown in Table 1.

**TABLE 1:** Patient characteristics in total and propensity score‐matched cohorts.

	Total cohort (*N* = 27,643)			PS‐matched cohort (1:1) (*N* = 17,554)		
	De Ritis ratio < 1.04 (*n* = 17,297)	De Ritis ratio ≥ 1.04 (*n* = 10,346)	SMD	De Ritis ratio < 1.04 (*n* = 8777)	De Ritis ratio ≥ 1.04 (*n* = 8777)	SMD
Baseline characteristics						
Age (years)^a^	58 (51,65)	63 (55,70)	0.365	61 (54, 67)	62 (54, 68)	0.006
Male (%)^a^	11,295 (65.3)	4699 (45.4)	0.408	4373 (49.8)	4391 (50)	0.004
BMI (kg/m^2^)^a^	25.6 (23.4,27.8)	24.5 (22.3,26.8)	0.314	24.8 (22.8, 27)	24.8 (22.6, 27.1)	0.011
ASA classification (%)^a^			0.135			0.009
I	945 (5.5)	446 (4.3)		433 (4.9)	420 (4.8)	
II	13,706 (79.2)	7809 (75.5)		6746 (76.9)	6739 (76.8)	
III or IV	2646 (15.3)	2091 (20.2)		1598 (18.2)	1618 (18.4)	
Tobacco use (%)^a^	2426 (14)	969 (9.4)	0.145	895 (10.2)	884 (10.1)	0.004
Alcohol use (%)^a^	2818 (16.3)	1206 (11.7)	0.134	1113 (12.7)	1072 (12.2)	0.014
Hypertension (%)	7255 (41.9)	4340 (41.9)	< 0.001	3884 (44.3)	3557 (40.5)	0.075
CHD (%)	1565 (9)	1039 (10)	0.034	901 (10.3)	840 (9.6)	0.023
Heart failure (%)	31 (0.2)	25 (0.2)	0.014	16 (0.2)	18 (0.2)	0.005
Myocardial infarction (%)	199 (1.2)	139 (1.3)	0.017	111 (1.3)	117 (1.3)	0.006
Arrhythmia (%)	2573 (14.9)	1608 (15.5)	0.019	1319 (15)	1305 (14.9)	0.004
COPD (%)	156 (0.9)	124 (1.2)	0.029	82 (0.9)	106 (1.2)	0.027
Renal insufficiency (%)	221 (1.3)	222 (2.1)	0.067	142 (1.6)	165 (1.9)	0.02
Peripheral vascular disease (%)	923 (5.3)	641 (6.2)	0.037	538 (6.1)	522 (5.9)	0.008
Malignant tumor (%)	8728 (50.5)	5580 (53.9)	0.07	4438 (50.6)	4684 (53.4)	0.056
Previous ischemic stroke (%)	902 (5.2)	627 (6.1)	0.037	516 (5.9)	508 (5.8)	0.004
Hepatitis virus carrier (%)	896 (5.2)	507 (4.9)	0.013	417 (4.8)	449 (5.1)	0.017
Preoperative FPG (mmol/L)^a^	6.44 (5.3,8.06)	5.86 (4.87,7.28)	0.233	6.13 (5.13,7.5)	5.96 (4.92,7.41)	0.019
Preoperative Hb (g/L)^a^	138 (126,149)	128 (117,139)	0.547	131 (120, 141)	131 (120, 141)	0.007
Preoperative platelet (g/L)	212 (174,256)	213 (173,260)	0.013	216 (176, 262)	212 (172, 257)	0.077
Preoperative ALB (g/L)^a^	41.4 (39,43.8)	40.4 (37.8,42.9)	0.262	40.8 (38.3, 43.1)	40.7 (38.2, 43.1)	0.002
Preoperative TBIL (μmol/L)^a^	11.2 (8.4,15.2)	10.2 (7.8,13.6)	0.147	10.4 (7.8, 14.1)	10.5 (8, 14)	0.002
Preoperative insulin (%)	8980 (51.9)	5234 (50.6)	0.027	4561 (52)	4354 (49.6)	0.047
Preoperative anticoagulants (%)	1113 (6.4)	835 (8.1)	0.063	652 (7.4)	652 (7.4)	< 0.001
Intraoperative parameters						
Emergency surgery (%)^a^	305 (1.8)	349 (3.4)	0.102	231 (2.6)	233 (2.7)	0.001
Neurosurgery (%)	1596 (9.2)	786 (7.6)	0.059	773 (8.8)	713 (8.1)	0.025
Surgery length (min)	157 (109,225)	158 (110,220)	0.036	155 (108, 222)	158 (110, 222)	0.014
Crystalloids infusion (mL/kg/h)^a^	7.94 (5.92,10.59)	8.7 (6.48,11.6)	0.181	8.48 (6.34, 11.3)	8.43 (6.33, 11.2)	0.02
Colloids infusion (mL/kg/h)	2.56 (0, 3.88)	2.76 (0, 4.2)	0.093	2.76 (0.04, 4.2)	2.78 (0, 4.21)	0.007
Intraoperative blood products (%)^a^	1920 (11.1)	1717 (16.6)	0.16	1267 (14.4)	1253 (14.3)	0.005

*Note:* Data are shown as median (IQR) for continuous variables and number (percentage) for categorical variables.

Abbreviations: ALB, albumin; ASA, American Society of Anesthesiologists; BMI, body mass index; CHD, coronary heart disease; COPD, chronic obstructive pulmonary disease; ENT, ear, nose, and throat; Hb, hemoglobin; IQR, interquartile range; PS, propensity score; SMD, standardized mean difference; TBIL, total bilirubin.

^a^ Variables matched in the PSM.

The odds of PIS were significantly increased in patients with a De Ritis ratio ≥ 1.04 than in those with a De Ritis ratio < 1.04 (2.55 [1.84–3.55]; *p* < 0.001). The odds of PIS for patients in the high De Ritis ratio group were still significantly increased after adjusting for baseline characteristics (2.25 [1.59–3.21]; *p* < 0.001), intraoperative parameters (2.50 [1.80–3.49]; *p* < 0.001), and all variables (2.29 [1.61–3.29]; *p* < 0.001) (Table 2). The detailed data from the crude analysis and multivariate models are shown in Table S2.

**TABLE 2:** Odds ratio for De Ritis ratio ≥ 1.04 for the risk of perioperative ischemic stroke in the total and propensity score‐matched cohorts.

	Odds ratio (95% CI)	95% CI	*p*
Logistic regression analysis (*n* = 27,643)			
Model 1 (univariable model)	2.55	1.84–3.55	< 0.001
Model 2 (baseline characteristics adjusted)	2.25	1.59–3.21	< 0.001
Model 3 (intraoperative parameters adjusted)	2.5	1.8–3.49	< 0.001
Model 4 (all confounding variables adjusted)	2.29	1.61–3.29	< 0.001
Propensity score analysis (*n* = 17,554)			
PS matched model (univariable model)	2.04	1.38–3.05	< 0.001

*Note:* Model 1 is a univariable model for crude analysis. Model 2 is a multivariable model including age, sex, ASA classification, tobacco use, alcohol use, hypertension, coronary heart disease, heart failure, myocardial infarction, peripheral vascular disease, previous ischemic stroke, preoperative fasting plasma glucose, preoperative hemoglobin, preoperative platelet, preoperative albumin, preoperative insulin, and preoperative anticoagulants. Model 3 is a multivariable model including surgical category, surgery length, urgency of surgery, and intraoperative blood products usage. Model 4 includes all the confounding variables.

Abbreviation: PS, propensity score.

### 3.3 Propensity Score Analysis

**FIGURE 2:**
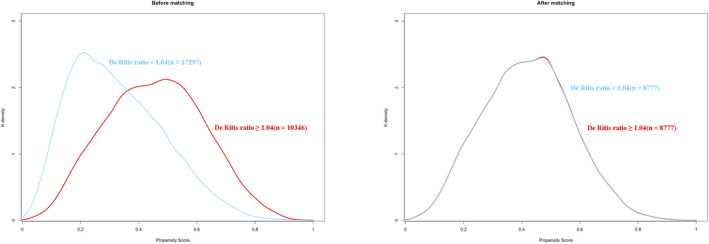
Propensity score distribution before and after propensity score matching.

We obtained 8777 patients in each group after PSM, with SMD for all variables less than 0.10 in the two groups (Table 1). The distribution of PS before and after matching in the low De Ritis ratio and high De Ritis ratio groups is graphically illustrated (Figure 2). PIS occurred in 0.42% (*n* = 37) of the patients in the low De Ritis ratio group and 0.85% (*n* = 75) of the patients in the high De Ritis ratio group in the PS‐matched cohort. In the PS‐matched cohort, the odds of PIS were significantly increased in the high De Ritis ratio group (2.04 [1.38–3.05]; *p* < 0.001) (Table 2). The detailed data for the PS‐matched cohort are presented in Table S3.

### 3.4 Sensitivity Analysis

The De Ritis ratio is related to the hepatitis viral carriage condition and alcohol usage, and for patients undergoing emergency surgery, preoperative transaminase levels may not reflect the actual physiological state. A series of sensitivity analyses was conducted by excluding hepatitis virus carriers, alcohol users, and those who underwent emergency surgery. The association between the De Ritis ratio and PIS remained significant even after the exclusion of hepatitis virus carriers (2.27 [1.59–3.27]; *p* < 0.001), alcohol users (2.47 [1.70–3.62]; *p* < 0.001), and those who underwent emergency surgery (2.34 [1.62–3.42]; *p* < 0.001) (Table 3).

**TABLE 3:** Sensitivity analysis of the association between De Ritis ratio and perioperative ischemic stroke.

	De Ritis ratio < 1.04 (PIS cases, *n*)	De Ritis ratio ≥ 1.04 (PIS cases, *n*) and PIS cases (*n*)	Odds ratio	95% CI	*p*
Hepatitis virus carriers excluded	16,401 (59)	9839 (89)	2.27	1.59–3.27	< 0.001
Alcohol users excluded	14,479 (52)	9140 (87)	2.47	1.7–3.62	< 0.001
Emergency surgery excluded	16,992 (55)	9997 (80)	2.34	1.62–3.42	< 0.001

### 3.5 Subgroup Analysis

**FIGURE 3:**
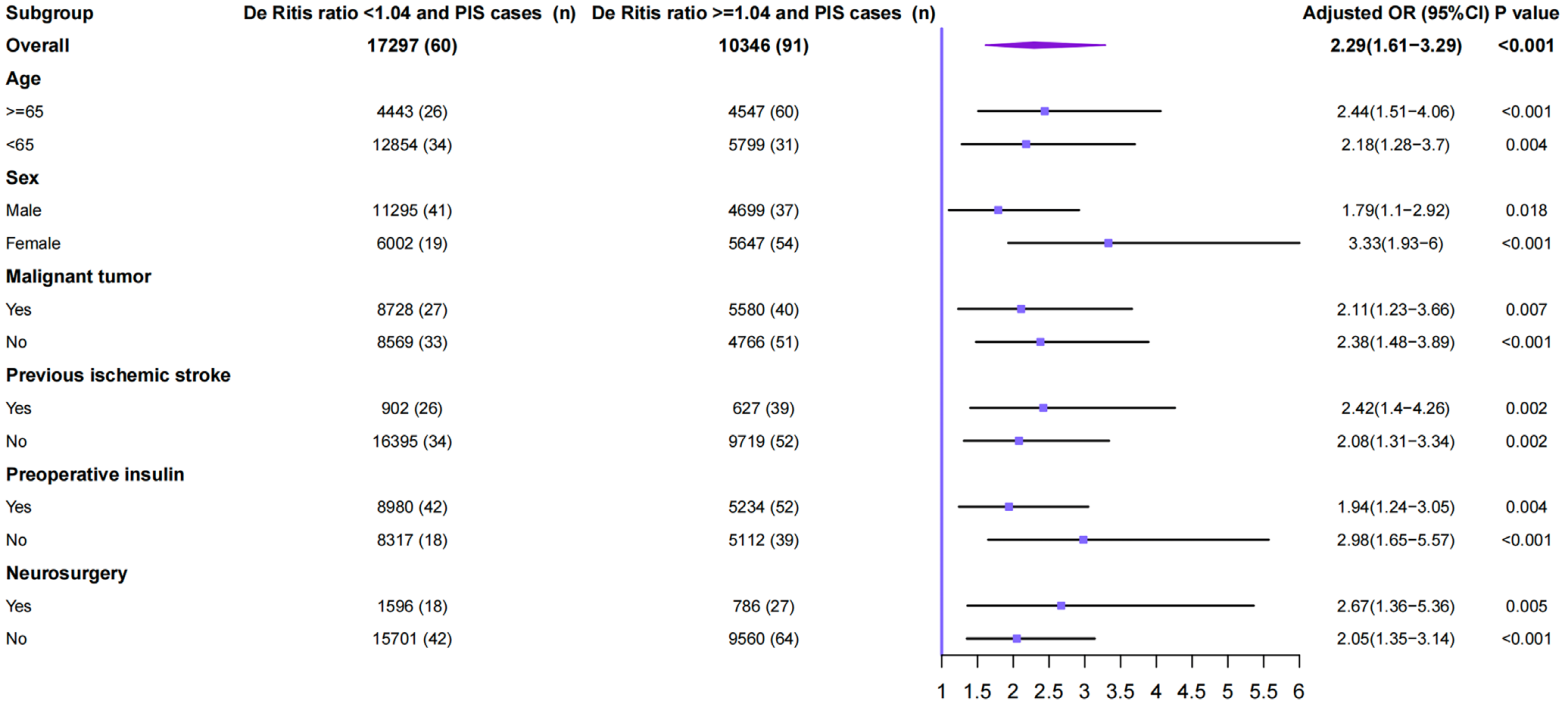
Subgroup analysis of the association between the De Ritis ratio and perioperative ischemic stroke (PIS).

We evaluated the association between the De Ritis ratio and PIS in subgroups of patients stratified by age, sex, malignant tumor, previous ischemic stroke, preoperative insulin medication, and surgical category. The OR of PIS was significant in subgroups stratified by age (≥ 65 years old: 2.44 [1.51–4.06]; *p* < 0.001; < 65 years old: 2.18 [1.28–3.70]; *p* = 0.004), sex (male: 1.79 [1.10–2.92]; *p* = 0.018; female: 3.33[1.93–6.00]; *p* < 0.001), malignant tumor (Yes: 2.11 [1.23–3.66]; *p* = 0.007; No: 2.38 [1.48–3.89]; *p* < 0.001), previous ischemic stroke (Yes: 2.42 [1.40–4.26]; *p* = 0.002; No: 2.08 [1.31–3.34]; *p* = 0.002), preoperative insulin medication (Yes: 1.94 [1.24–3.05]; *p* = 0.004; No: 2.98 [1.65–5.57]; *p* < 0.001), and surgical category (neurosurgery: 2.67 [1.36–5.36]; *p* = 0.005; non‐neurosurgery: 2.05 [1.35–3.14]; *p* < 0.001, Figure 3).

## 4 Discussion

The present study indicated that the De Ritis ratio is strongly associated with PIS risk in patients with type 2 DM during noncardiovascular surgeries. We demonstrated that type 2 diabetic patients with an elevated preoperative De Ritis ratio had a significantly higher PIS risk than patients with a relatively lower De Ritis ratio after adjusting for baseline characteristics and intraoperative parameters. The associations were consistently maintained in the PSM analysis, sensitivity analysis, and subgroups stratified by age, sex, malignant tumor, previous ischemic stroke, preoperative insulin medication, and surgical category. This predictive capacity of the De Ritis ratio might help with risk stratification during preoperative assessment and in identifying diabetic patients at high risk for PIS.

AST and ALT levels are routine laboratory tests for hepatic function evaluation at the time of hospital admission. Plasma AST and ALT levels are also considered to be involved in metabolism‐related hepatic physiology and pathophysiology beyond hepatic cell damage [20, 21]. Generally, ALT is thought to be specific to the liver and is more related to insulin resistance and type 2 DM [22] while AST also originates from other organs and is an indicator of ischemic injuries as well [23, 24, 25]. For patients with metabolic dysfunction or type 2 DM, the De Ritis ratio should be < 1 because the ALT level is generally elevated and is higher than the AST level in this population [14, 22]. The median De Ritis ratio in the current cohort was 0.93, which is in line with previous studies showing that the De Ritis ratio is inversely associated with type 2 DM [13, 14, 26].

The pathogenesis of type 2 DM involves metabolic syndrome and insulin resistance, resulting in increased oxidative stress, endothelial dysfunction, and atherosclerosis [27, 28, 29], which are closely related to an increased risk of ischemic stroke. The mechanisms by which an elevated De Ritis ratio affects PIS risk in patients with DM are still obscure. Serum aminotransferase alterations are frequently observed in patients with DM and are associated with metabolic dysfunction and insulin resistance [14, 22, 30]. Elevation of ALT levels in the circulation may not necessarily indicate hepatic cell damage in diabetic patients. One hypothesis is that the elevated ALT levels are an adaptive response to the increased hepatic metabolic demands to maintain the steady state of glucose and amino acid metabolism [20, 21]. We assumed that with the progression of the diabetes course, ALT activity might be altered at different levels, and a reversed change in the De Ritis ratio in diabetic patients may indicate potential decompensation of hepatic function.

Previous studies have verified that a De Ritis ratio of < 1 is a strong predictor of NAFLD, and a reversed De Ritis ratio of > 1 is a significant indication of hepatic fibrosis or even cirrhosis [12, 15, 31]. NAFLD is a common accompanying condition in patients with type 2 DM, and the reported prevalence of NAFLD in diabetic patients is up to 70% [32]. Notably, diabetes also increased the risk of hepatic fibrosis in patients with NAFLD, even when the aminotransferase levels were within the normal range [33]. In addition, hepatic fibrosis has been proven to be independently associated with atherosclerosis in the carotid artery, even when no apparent clinical evidence of hepatic disease is found [34]. Considering these points, we speculated that a potential hepatic fibrosis led to an exacerbated PIS risk in patients with type 2 DM. An elevated De Ritis ratio may not be a “cause” of PIS, but a reversed De Ritis ratio of > 1 in patients with type 2 DM can be regarded as strong evidence for potential hepatic fibrosis. Furthermore, it is noteworthy that coagulation activation plays an important role in the pathogenesis of hepatic fibrosis [35, 36]; thus, an elevated De Ritis ratio in type 2 diabetic patients might indicate hypercoagulability and thrombogenic diathesis, which subsequently predisposes diabetic patients to PIS under surgical stress. Because a reversed De Ritis ratio of > 1 was related to hepatic fibrosis or even cirrhosis, the relationship between the De Ritis ratio and PIS might be to the result of asymptomatic hepatic dysfunction or fibrosis.

The crucial role of inflammation in the pathogenesis of DM and atherosclerosis should also be considered. The production of intravascular reactive oxygen species was enhanced during chronic hyperglycemia, resulting in vascular oxidative stress and inflammatory pathway activation. Increased inflammation accelerates atherosclerotic plaque formation and thus correlates with macro‐ and microvascular complications in diabetic individuals [37]. In a prospective cohort study of 402 patients with type 2 DM, the De Ritis ratio was positively correlated with serum inflammatory cytokines and was an independent predictor of diabetic nephropathy [19]. This finding indicated that the elevated De Ritis ratio in diabetic patients might represent increased inflammatory activity, which was also associated with increased risk of PIS in surgical patients. Additionally, as patients with an elevated De Ritis ratio were inclined to have a thrombogenic diathesis, perioperative inflammatory responses may also facilitate thromboembolic formation [38, 39] and subsequently increase the risk of PIS.

In recent years, the De Ritis ratio has also been used as a prognostic predictor of ischemic injuries. In a cohort of 1355 patients with acute MI, a De Ritis ratio > 1.2 was determined to be a high risk for long‐term mortality [16]. In a small cohort of 374 patients with cardiac arrest, a higher De Ritis ratio at baseline was associated with increased mortality and unfavorable neurological outcomes [17]. In addition, in a cohort of 421 subjects with acute ischemic stroke, an increased De Ritis ratio was associated with poor outcomes at 3 months after stroke [18]. The current study revealed a robust association between preoperative De Ritis ratio and PIS. Our results add to these findings that an elevated De Ritis ratio may also be relevant to PIS risk in patients with type 2 DM. In a series of sensitivity and subgroup analyses, this association remained essentially unaltered. Plasma aminotransferase levels were gender related [40] and also associated with advancing age [41] and malignant tumors [42, 43]. Previous stroke, DM requiring insulin therapy, and neurosurgeries have been validated as strong risk factors for PIS [4, 6]. Considering these correlations, we subdivided the participants according to age, sex, malignant tumor, previous ischemic stroke, preoperative insulin medication, and surgical category to validate the associations further. These associations were consistently maintained in the sensitivity and subgroup analyses.

This retrospective cohort study had several notable strengths. First, to the best of our knowledge, the role of the De Ritis ratio in PIS risk prediction in type 2 diabetic patients undergoing noncardiovascular surgery has not been evaluated. Second, various multivariate models and PSM analyses were conducted to minimize potential bias. Third, we performed a series of sensitivity and subgroup analyses to validate the consistency of the findings. Although the mechanisms by which elevated De Ritis ratio increases the risk of PIS have not yet been identified, potential hepatic dysfunction and enhanced inflammation activation might explain the role of the De Ritis ratio in the pathology of PIS in the type 2 diabetic population.

However, this study had several limitations. First, the enrolled patients were from a single center; thus, generalization of these findings may be restricted. Second, this is a retrospective cohort study, and potential confounding factors may not be entirely ruled out; thus, well‐designed RCT research is required to validate our conclusion. Third, as neither liver biopsy nor ultrasound was performed in this cohort, it is difficult to ascertain whether the relationship between the De Ritis ratio and PIS is specifically liver related or due to other pathogenesis, and further studies are required to elucidate the exact mechanisms. Nonetheless, we demonstrated a strong association between the preoperative De Ritis ratio and PIS risk in patients with type 2 DM. These findings may have clinical implications for preoperative assessment, as patients with an elevated De Ritis ratio may have a higher risk of PIS and thus deserve closer perioperative monitoring. Although our study was limited by the unavailability of diagnostic examinations, considering the benefits and cost‐effectiveness, the De Ritis ratio may be a promising predictive biomarker for PIS risk evaluation in patients with type 2 DM undergoing noncardiovascular surgeries.

## 5 Conclusions

The main conclusion of the current study is that the preoperative De Ritis ratio is an independent prognostic factor of PIS in patients with type 2 DM undergoing noncardiovascular surgery. Although the exact mechanisms have not been clarified, as the De Ritis ratio may provide additional information on PIS risk assessment of patients with type 2 DM, it may act as a novel biochemical indicator for PIS prediction.

## Author Contributions

S.L. and B.W. wrote the manuscript with contributions from all authors. W.M., Y.M., and S.L. designed the study. L.M., H.Y., M.L., Y.S., J.L., Z.Z., J.C., and Y.L. were responsible for data extraction and acquisition. W.M. and S.L. designed and conducted the statistical analyses.

## Ethics Statement

The studies involving human participants were reviewed and approved by the Medical Ethics Committee of the First Medical Center of Chinese PLA General Hospital. The requirement for written informed consent for participation was waived in accordance with national legislation and institutional requirements.

## Conflicts of Interest

The authors declare no conflicts of interest.

## Supporting information


Data S1.

## Data Availability

The data analyzed in this study are available from the corresponding author upon reasonable request.
