# Supplementary material for: The Association Between Aspartate Transaminase to Alanine Transaminase Ratio and Perioperative Ischemic Stroke in Patients With Diabetes: A Retrospective Cohort Study
Source: CNS Neurosci Ther. 2025 Jan 21;31(1):e70223. doi: 10.1111/cns.70223 (PMC11751254; doi:10.1111/cns.70223)
Supplement: Supplementary file 1 — Data S1. [file CNS-31-e70223-s001.docx]

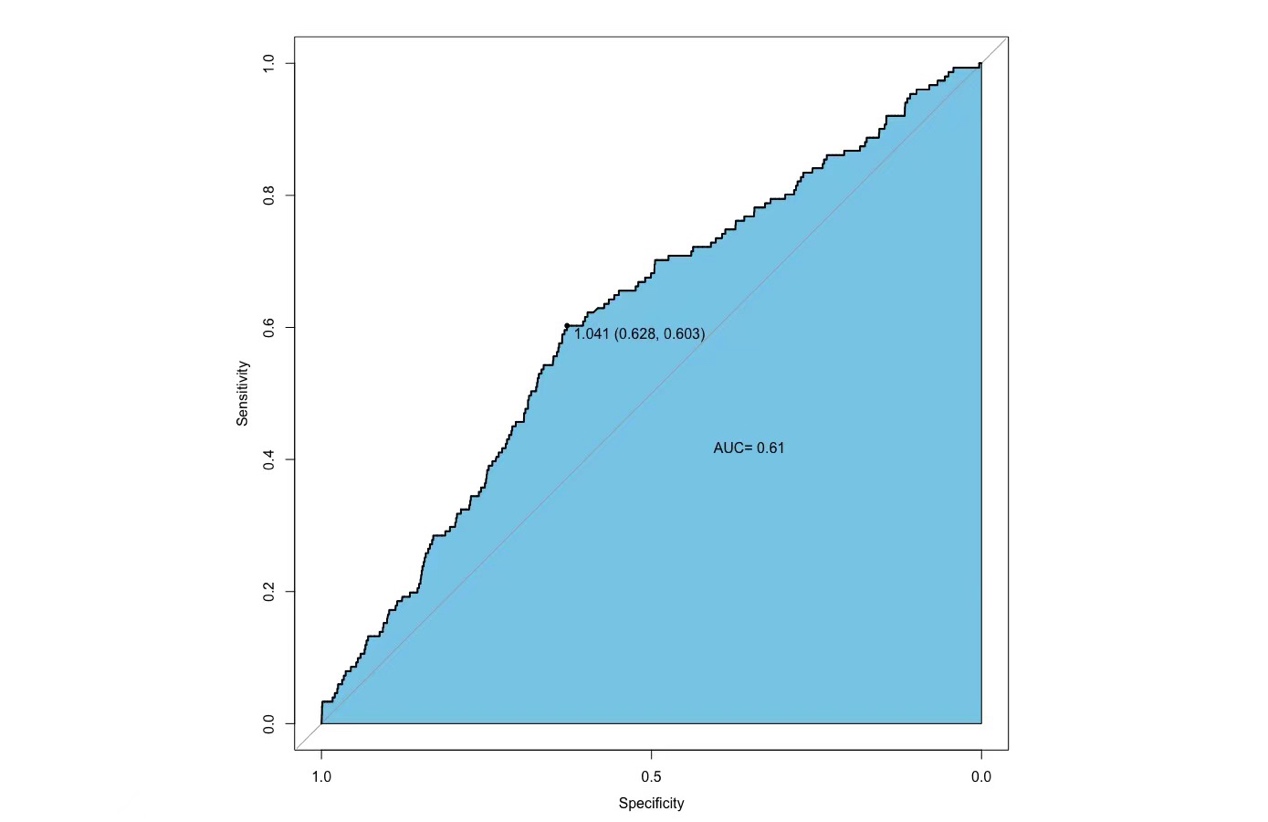


Figure S1 ROC curve of De Ritis ratio for perioperative ischemic stroke.

ROC, receiver operating characteristics curve; AUC, area under curve.

Supplementary Table 1: ICD-9/10 Diagnosis Codes for Ischemic Stroke

| Ischemic stroke | ICD-9/ICD-10 | 433.X1/I63.X | Occlusion and stenosis of precerebral arteries with cerebral infarction |
| --- | --- | --- | --- |
|  | ICD-9 | 434.X1 | Occlusion of cerebral arteries with cerebral infarction |
|  | ICD-9/ICD-10 | 437.1/I67.81,  I67.89 | Other generalized ischemic cerebrovascular disease |
|  | ICD-9/ICD-10 | 437.9/I67.9 | Unspecified cerebrovascular disease |

Supplementary Table 2 Univariate and Multivariate Logistic Regression Analysis for Perioperative Stroke in Model 4

| Variables | Univariate analysis |  | Multivariate analysis |  |
| --- | --- | --- | --- | --- |
|  | OR (95% CI) | P value | OR (95% CI) | P value |
| De Ritis ratio ≥ 1.04 (Yes vs No) | 2.549 (1.844-3.55) | < 0.001 | 2.292 (1.607-3.288) | <0.001 |
| Age | 1.053 (1.037–1.07) | <0.001 | 1.035 (1.017-1.054) | < 0.001 |
| Sex (female vs male) | 1.287 (0.933-1.772) | 0.123 | 0.969 (0.669-1.406) | 0.868 |
| BMI | 1.008 (0.965–1.052) | 0.711 |  |  |
| ASA classification  Class I | reference |  |  |  |
| Class II | 1.878 (0.704–7.658) | 0.283 | 1.19(0.431-4.934) | 0.772 |
| Class III or IV | 6.036 (2.234–24.743) | 0.002 | 1.478(0.507-6.308) | 0.529 |
| Tobacco use (Yes vs No) | 0.505(0.249-0.911) | 0.037 | 0.6(0.274-1.186) | 0.168 |
| Alcohol use (Yes vs No) | 0.505(0.265-0.873) | 0.023 | 0.689(0.336-1.298) | 0.276 |
| Hypertension (Yes vs No) | 2.293 (1.655-3.206) | < 0.001 | 1.259 (0.877-1.817) | 0.214 |
| CHD (Yes vs No) | 2.4(1.578-3.536) | < 0.001 | 1.365 (0.85-2.127) | 0.182 |
| Heart failure (Yes vs No) | 6.82 (1.11-22.172) | 0.008 | 1.785 (0.256-7.201) | 0.478 |
| Myocardial infarction (Yes vs No) | 2.793 (0.986-6.173) | 0.025 | 1.134(0.373-2.782) | 0.802 |
| Arrhythmia (Yes vs No) | 1.394 (0.917-2.052) | 0.104 |  |  |
| COPD (Yes vs No) | 0.65(0.037-2.911) | 0.669 |  |  |
| Renal insufficiency (Yes vs No) | 2.115(0.747-4.666) | 0.101 |  |  |
| Peripheral vascular disease (Yes vs No) | 5.929 (4.056-8.487) | < 0.001 | 2.05 (1.32-3.124) | 0.001 |
| Malignant tumor (Yes vs No) | 0.742 (0.536-1.023) | 0.069 |  |  |
| Previous ischemic stroke (Yes vs No) | 13.437 (9.666-18.593) | < 0.001 | 7.123 (4.898-10.31) | < 0.001 |
| Hepatitis virus carrier (Yes vs No) | 0.378(0.093-0.997) | 0.095 |  |  |
| Preoperative FPG | 1.101(1.052-1.147) | < 0.001 | 1.059(1.003-1.113) | 0.03 |
| Preoperative hemoglobin | 0.99 (0.981-0.998) | 0.013 | 1.008(0.997-1.019) | 0.151 |
| Preoperative platelet | 1.004(1.002-1.005) | < 0.001 | 1.003(1.001-1.005) | < 0.001 |
| Preoperative albumin | 0.942 (0.909-0.978) | 0.001 | 0.985(0.942-1.03) | 0.504 |
| Preoperative total bilirubin | 0.999(0.994-1.003) | 0.736 |  |  |
| Preoperative Insulin (Yes vs No) | 1.562(1.127-2.183) | 0.008 | 1.27(0.892-1.82) | 0.188 |
| Preoperative anticoagulants (Yes vs No) | 2.387 (1.49-3.653) | < 0.001 | 1.016(0.602-1.649) | 0.952 |
| Emergency surgery (Yes vs No) | 4.989(2.843-8.167) | < 0.001 | 2.296 (1.173-4.241) | 0.011 |
| Neurosurgery (Yes vs No) | 4.57 (3.187-6.446) | < 0.001 | 3.686(2.386-5.623) | < 0.001 |
| Surgery length | 1.002 (1.001–1.003) | < 0.001 | 1.002 (1-1.003) | 0.012 |
| Crystalloid infusion | 0.962 (0.92-1.002) | 0.073 |  |  |
| Colloid infusion | 1.022(0.953-1.092) | 0.528 |  |  |
| Intraoperative blood products (Yes vs No) | 1.782(1.185-2.602) | 0.004 | 1.294 (0.806-2.029) | 0.273 |

Abbreviations: ASA, American Society of Anesthesiologists; BMI, body mass index; CHD, coronary heart disease; COPD, chronic obstructive pulmonary disease.

Supplementary Table 3 Univariate Logistic Regression Analysis for Perioperative Stroke in the PS Matched Cohort.

| Variables | Univariate analysis |  |
| --- | --- | --- |
|  | OR (95% CI) | P value |
| De Ritis ratio ≥ 1.04 (Yes vs No) | 2.036 (1.382-3.052) | <0.001 |
| Age | 1.042 (1.023–1.062) | <0.001 |
| Sex (female vs male) | 0.962 (0.662-1.396) | 0.837 |
| BMI | 1.058 (1.005–1.112) | 0.028 |
| ASA classification  Class I | reference |  |
| Class II | 2.061 (0.645-12.569) | 0.314 |
| Class III or IV | 6.038 (1.863-37.041) | 0.013 |
| Tobacco use (Yes vs No) | 0.774(0.363-1.447) | 0.462 |
| Alcohol use (Yes vs No) | 0.613(0.288-1.146) | 0.16 |
| Hypertension (Yes vs No) | 1.887 (1.299-2.764) | <0.001 |
| CHD (Yes vs No) | 1.986 (1.188-3.158) | 0.006 |
| Heart failure (Yes vs No) | 9.892 (1.591-33.153) | 0.002 |
| Myocardial infarction (Yes vs No) | 2.106 (0.516-5.639) | 0.206 |
| Arrhythmia (Yes vs No) | 1.394 (0.852-2.183) | 0.164 |
| COPD (Yes vs No) | 0.831(0.047-3.748) | 0.854 |
| Renal insufficiency (Yes vs No) | 2.095(0.639-5.029) | 0.149 |
| Peripheral vascular disease (Yes vs No) | 4.319 (2.681-6.697) | < 0.001 |
| Malignant tumor (Yes vs No) | 0.717 (0.491-1.041) | 0.082 |
| Previous ischemic stroke (Yes vs No) | 12.186 (8.287-17.787) | < 0.001 |
| Hepatitis virus carrier (Yes vs No) | 0.349 (0.058-1.1) | 0.14 |
| Preoperative FPG | 1.133(1.073-1.188) | < 0.001 |
| Preoperative hemoglobin | 1.001 (0.99-1.012) | 0.895 |
| Preoperative platelet | 1.003(1.001-1.005) | 0.004 |
| Preoperative albumin | 0.991 (0.948-1.038) | 0.711 |
| Preoperative total bilirubin | 0.998(0.987-1.004) | 0.644 |
| Preoperative Insulin (Yes vs No) | 1.446(0.994-2.125) | 0.056 |
| Preoperative anticoagulants (Yes vs No) | 2.09 (1.183-3.456) | 0.007 |
| Emergency surgery (Yes vs No) | 4.511 (2.337-7.937) | < 0.001 |
| Neurosurgery (Yes vs No) | 4.597 (3.013-6.858) | < 0.001 |
| Surgery length | 1.002 (1–1.004) | 0.008 |
| Crystalloid infusion | 0.958 (0.91-1.006) | 0.095 |
| Colloid infusion | 0.984 (0.906-1.065) | 0.704 |
| Intraoperative blood products (Yes vs No) | 1.812(1.144-2.773) | 0.008 |

Abbreviations: ASA, American Society of Anesthesiologists; BMI, body mass index; CHD, coronary heart disease; COPD, chronic obstructive pulmonary disease.
